# Mechanisms of cardiac iron homeostasis and their importance to heart function

**DOI:** 10.1016/j.freeradbiomed.2018.08.010

**Published:** 2019-03

**Authors:** Samira Lakhal-Littleton

**Affiliations:** Department of Physiology, Anatomy and Genetics, University of Oxford, Parks Road, Oxford OX1 3PT, United Kingdom

**Keywords:** Iron, Homeostasis, Heart, Hepcidin, Ferroportin

## Abstract

Heart disease is a common manifestation in conditions of iron imbalance. Normal heart function requires coupling of iron supply for oxidative phosphorylation and redox signalling with tight control of intracellular iron to below levels at which excessive ROS are generated. Iron supply to the heart is dependent on systemic iron availability which is controlled by the systemic hepcidin/ferroportin axis. Intracellular iron in cardiomyocytes is controlled in part by the iron regulatory proteins IRP1/2. This mini-review summarises current understanding of how cardiac cells regulate intracellular iron levels, and of the mechanisms linking cardiac dysfunction with iron imbalance. It also highlights a newly-recognised mechanism of intracellular iron homeostasis in cardiomyocytes, based on a cell-autonomous cardiac hepcidin/ferroportin axis. This new understanding raises pertinent questions on the interplay between systemic and local iron control in the context of heart disease, and the effects on heart function of therapies targeting the systemic hepcidin/ferroportin axis.

## Highlights

• Iron is essential for normal heart function but excess iron is detrimental to it.
• Cardiomyocyte iron levels are a balance between uptake and release.
• Cardiomyocyte ferroportin is essential for iron release, and for normal cardiac function.
• Hepcidin produced by cardiomyocytes regulates local iron release.
• The cardiac hepcidin/ferroportin axis is essential for heart function.

## Graphical abstract

**Figure:**
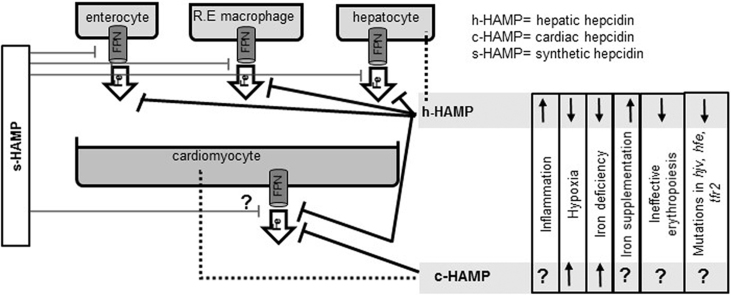

## 1 Principles of iron homeostasis

The average amount of iron in a human male is ~4 g, of which ~2.3 g is present in the red blood cell compartment and ~3 mg complexed to transferrin (Tf-Fe^3+^) in the plasma [1]. The rest is present intracellularly within peripheral tissues. Inside cells, most of the iron is complexed to protein chaperones such as ferritin, or present in functional groups; e.g. heme prosthetic or iron-sulfur groups [2], [3], [4] The rest represents the chelatable labile iron pool (LIP), the size of which varies between different cell types [5].

Cells must maintain an adequate supply of iron for iron-dependent processes, while at the same time restricting the size of LIP to prevent excessive ROS generation from Fenton-type reactions [6]. Cellular iron homeostasis is achieved through control of iron uptake by transferrin receptor 1 (TfR1) and divalent metal transporter 1 (DMT-1); iron storage by ferritin; iron utilisation in heme synthesis by erythroid 5-aminolevulinic acid synthase (ALAS2); and in some cells, iron export by ferroportin (SLC40A1 or FPN) [7], [8]. These processes are orchestrated by the iron regulatory proteins, IRP1 and IRP2. Their activity involves an iron-sensing step, whereby high intracellular iron reduces the RNA-binding ability of IRP1 and the stability of IRP2. The second step, which occurs preferentially under conditions of low iron, involves the binding of IRP1 and IRP2 at iron regulatory elements IREs, either at the 5′UTR to cause translational repression (e.g ferroportin, L-ferritin, H-ferritin, Alas2) or at the 3′UTR to increase transcript stability (e.g TfR1, Dmt1) [7], [8].

The body must maintain adequate supply of iron for key physiological and developmental processes such as erythropoiesis, bone growth and neuronal development. This supply is achieved primarily from the recycling of iron from senescent red blood cells in the reticuloendothelial macrophages [8], [9]. Other sources of iron are enterocytes (the site of dietary iron absorption), and hepatocytes (the site of iron storage) [10], [11]. Systemic iron homeostasis is orchestrated by the hepcidin/ferroportin axis. Hepcidin is the liver-derived hormone that controls systemic iron availability through the binding, and internalisation of ferroportin. Ferroportin is the only known mammalian iron export protein, and enables the release of iron into the circulation from the sites of iron recycling, absorption and storage [10], [11]. Genetic mutations that impair hepcidin production or binding to ferroportin are associated with systemic iron overload (primary hemochromatosis) [12], while mutations in negative regulators of hepcidin (matriptase-2) cause iron refractory iron deficiency anaemia (IRIDA) due to inappropriately high hepcidin levels [13]. As the major site of iron demand in the body, the bone marrow exerts dominant control over hepcidin production. Hepcidin production in the liver is reduced by the endocrine action of erythroferrone derived from the stimulated erythroid compartment [14]. This explains the secondary iron overload associated with ineffective erythropoiesis (e.g. β-thalassemia) [15]. Hepcidin production is also suppressed by hypoxia (possibly in a manner dependent on erythroferrone) and stimulated by high transferrin saturation and by inflammation [16]. The anaemia of chronic disease is attributed to the stimulation of hepcidin by inflammation [17].

## 2 The importance of iron control in the heart

The average humy weight iron predominantly present in ferritin and within the mitochondrial compartment [18], [19]. The concentration of ferritin iron within the heart increases gradually with age while that of mitochondrial iron increases rapidly during growth and remains relatively stable in the adult heart [20]. Mitochondrial iron is present mainly in iron-sulfur clusters and heme functional groups [20]. Iron-mediated oxidation-reduction reactions are essential to the metabolism of oxygen in the heart. Iron in iron-sulfur and heme groups is required for electron transfer and oxygen activation in oxidative phosphorylation [2], [3], while labile free iron is required for oxygen activation by dioxygenases [21], and as a catalyst for redox signalling [22]. At the same time, this reactivity with oxygen underpins the ability of free iron to participate in Fenton-type reactions, producing ROS which are damaging to proteins and lipids [6]. The high rate of oxygen turnover in the heart necessitates a fine balance between, on the one hand, adequate supply of iron for the synthesis of iron-sulfur and heme groups, and on the other hand, tight control of the size of LIP to below levels at which excessive ROS are generated.

The requirement for such tight control of cardiac iron levels may also explain why heart failure is a common denominator in conditions of systemic iron imbalance. Iron overload cardiomyopathy is the leading cause of death in primary iron overload and an important co-morbidity in secondary iron overload [23], [24]. It manifests initially as restrictive cardiomyopathy with diastolic dysfunction, and often progresses to dilated cardiomyopathy [25], [26], [27]. In the setting of iron overload, where circulating non-transferrin bound iron (NTBI) levels are high, Fe^+2^ is taken up into cardiomyocyte through LTCCs; a route of uptake that is not coupled to the sensing of size of LIP, because LTCCs are not regulated by IRPs [27]. Additionally, the fact that the heart is affected first in these patients, despite the degree of iron loading in the heart being much lower than that of the liver, indicates that cardiomyocytes have greater sensitivity than hepatocytes to changes in LIP. This idea is supported by the finding that, under normal conditions, the concentration of non-ferritin non-heme Fe^2+^in the heart is considerably lower than that of the liver [20]. Beyond the cell damage caused by ROS, there is mounting evidence that excess intracellular iron promotes ferroptosis, a form of regulated cell death driven by iron-dependent lipid peroxidation [28]. In the myocardium, increased LIP, either in the setting of hemochromatosis, or following cardiac haemorrhage, could promote ferroptosis, thereby contributing to cardiac cell death [29]. Finally, there is evidence that increased LIP, and in particular free Fe^2+^, impinges directly on excitation-contraction coupling in cardiomyocytes, which may account for the diastolic dysfunction seen in the early stages of disease [25], [26], [27].

Another form of iron imbalance is iron deficiency. It is the most widespread nutritional disorder worldwide, including in industrialised nations, where it commonly co-exists with cardiovascular disease [30]. It is formally a recognised co-morbidity in chronic heart disease and acute heart failure [31], [32]. In heart failure, iron deficiency is highly prevalent, with some studies reporting it in 50% of patients in their cohorts [33], [34]. Depending on the aetiology, iron deficiency is either absolute (e.g., due to blood loss, malabsorption due gastrointestinal abnormalities) or functional, where iron is sequestered from the circulation (e.g., due to effect of inflammation on hepcidin levels). Importantly, iron deficiency, independently of anaemia, has been found to be a predictor of mortality, adverse cardiovascular events and quality of life [31], [32], [35]. In the last decade, a number of randomized trials have provided unequivocal evidence for the benefits of iron supplementation in patients with chronic heart failure. While oral iron supplementation has had limited success (partly due to limited absorption particularly in patients with inflammation), intravenous iron preparations such as ferric carboxymaltose and iron sucrose have been shown to improve a number of outcomes in patients with heart failure, including 6-min walk test, self-reported Patient Global Assessment, exercise capacity, hospitalisation due to cardiovascular events and mortality (summarised in 35). The European Society of Cardiology's guidelines now recommend intravenous iron replacement in chronic heart failure [36]. Less clear are the benefits of iron supplementation in acute heart failure, where there is a concern that such treatment could exacerbate ischemia reperfusion injury.

**Fig. 1:**
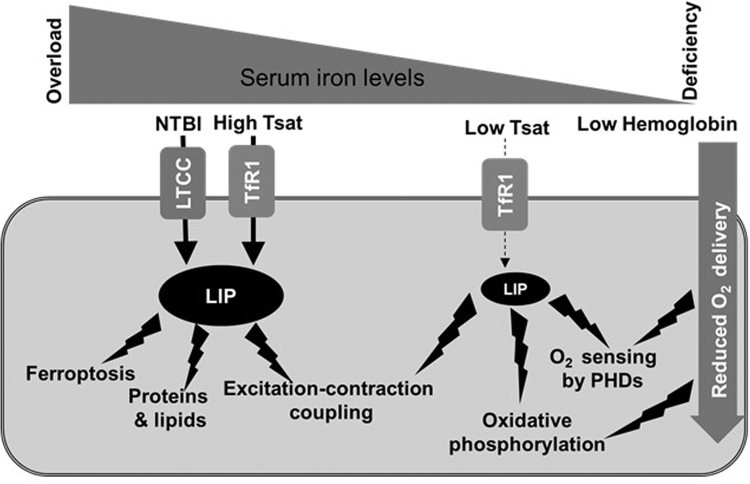
Effects of systemic iron imbalance on the cardiomyocyte. In conditions of iron overload, transferrin saturation (Tsat) is elevated, and non-transferrin bound iron NTBI increases in the circulation. NTBI (in the form of Fe ^2+^) is taken up into cardiomyocytes by L-type calcium channels (LTCC), which are not regulated in response to intracellular iron overload. Higher levels of iron uptake increase the size of the labile iron pool (LIP) to levels that generate excessive reactive oxygen species (ROS). These damage proteins and lipids and interfere with excitation-contraction coupling, leading to diastolic dysfunction. In addition, increased LIP could promote ferroptosis following injury (e.g. ischemia reperfusion injury, cardiac haemorrhage). In conditions of iron deficiency, the iron available for uptake by cardiomyocytes is reduced because of lower Tsat. This results in a reduction in the size of LIP, limiting the availability of iron for the synthesis of enzymes involved in oxidative phosphorylation. Other effects may also include impaired redox signalling and oxygen sensing by HIF prolyl hydroxylases, which utilise iron as a co-factor. When iron deficiency is accompanied by anaemia, lower haemoglobin levels can result in reduced oxygen delivery to the cardiomyocyte. This in turn also affects oxygen sensing and oxidative phosphorylation.

The mechanisms underlying the detrimental effects of iron deficiency in heart failure are only beginning to be explored. In part, iron deficiency anaemia affects the heart by reducing muscle exercise capacity, and limiting oxygen availability for use in oxidative phosphorylation within cardiomyocytes [37], [38]. However, anaemia is estimated to occur only in ~25% of iron-deficient individuals [39], [40], [41]. In the context of chronic heart failure, clinical outcomes are worse in non-anaemic iron-deficient patients than in anaemic iron-replete patients, suggesting that iron deficiency per se affects the heart directly, and in a manner that is distinct from the effects of anaemia [35]. Other than its requirement for haemoglobin synthesis, iron is essential for metabolic and signalling processes. In the heart, recent evidence is pointing towards a direct effect of intracellular iron levels within the cardiomyocyte on cardiac function. In one mouse model lacking cardiomyocyte TfR1, severely reduced iron levels in the cardiomyocytes resulted in fatal heart failure by the second week of age, in part due to failure of mitochondrial respiration [42]. The severe and early nature of the phenotype seen in these mice likely reflects the previously recognised need to rapidly increase mitochondrial iron levels during growth [20]. In another mouse model of dysregulated cardiac iron export (discussed in more detail in the next section), more progressive depletion of cardiomyocyte iron resulted in heart failure developing between 3 and 6 months of age [43]. In that setting, the activity of the electron transport chain was also reduced. One pertinent question is what pathways, other than mitochondrial respiration, are affected by intracellular iron deficiency in the heart. Pathways of interest include oxygen sensing by Hypoxia-inducible factor (HIF) prolyl hydroxylases (PHDs which require iron as a co-factor) and redox signalling which controls excitation-contraction coupling in the cardiomyocyte [25], [44]. Fig. 1 summarises the effects of systemic iron imbalance on the cardiomyocyte.

## 3 New mechanisms of iron control in the heart

Like other cells in the body, cardiomyocytes acquire iron predominantly by uptake of transferrin through TfR1. The importance of TfR1 for cardiac function is supported by the finding that mice lacking cardiac TfR1 manifest severe heart failure [42]. Inside the cell, iron that is not utilised in the synthesis of heme and iron-sulfur clusters is stored in ferritin. The levels of both TfR1 and ferritin are regulated by the IRP/IRE system [7]. The importance of IRP1 and 2 in the heart has been demonstrated in a mouse model with cardiomyocyte-specific deletion of the *Irp1* and *Irp2* genes. Such mice have an impaired left ventricular response to dobutamine challenge and an exacerbated injury following myocardial infarction [45].

One feature of cardiomyocytes is that they also express relatively high levels of ferroportin and hepcidin, despite having no role in systemic iron control [43], [46]. The functions of cardiac hepcidin and ferroportin have been explored recently. It was shown that mice with a cardiomyocyte-specific deletion of the ferroportin gene develop fatal left ventricular dysfunction by three months of age. The dysfunction was caused by a three-fold increase in iron levels within cardiomyocytes, and was prevented when animals were fed an iron-deficient diet. Notably, downregulation of TfR1, an IRP-driven response to increased cardiomyocyte iron content, was not sufficient to prevent iron overload in ferroportin-deficient hearts, demonstrating that ferroportin-mediated iron release is an essential component of cardiomyocyte iron homeostasis [46]. Comparison between mice lacking cardiomyocyte ferroportin and a mouse model of hemochromatosis (ubiquitous deletion of hepcidin) yielded interesting insights into the pathophysiology of iron overload. It was found that the total quantity of iron in the ferroportin-deficient hearts was much lower than in hemochromatosis hearts. Nonetheless, fatal heart failure occurred in the former but not the latter setting. Closer examination of the distribution of iron showed that in the ferroportin-deficient heart, iron was preferentially retained within the cardiomyocytes, whereas in the hemochromatosis model, most of the iron was outside of the cardiomyocytes, consistent with the marked upregulation of cardiomyocyte ferroportin in this model. This comparison demonstrated that ferroportin in cardiomyocytes controls the site of deposition of iron in the heart in the setting of systemic iron overload, and thereby determines the severity with which iron deposition affects cardiac function [46]. Beyond control of iron export in the steady state, it would be important to establish how the levels of cardiac ferroportin affect iron-dependent processes that are important in the disease state, e.g. ferroptosis [28], [29].

The function of cardiac hepcidin was interrogated using two approaches [43]. First, cardiomyocyte-specific deletion of hepcidin resulted in fatal left ventricular dysfunction in mice between three and six months of age, despite the maintenance of normal systemic iron levels. Second, animals with cardiomyocyte-specific knock-in of the ferroportin isoform C326Y, which retains its iron export function but loses its hepcidin binding, also developed heart failure of similar nature and timecourse to that seen in animals lacking cardiomyocyte hepcidin. In both settings, the cardiomyocytes were found to be iron-depleted due to increased ferroportin-mediated iron export. The development of cardiac dysfunction in cardiac-hepcidin knockouts was prevented by intravenous iron supplementation. Taken together, these results demonstrate that the cardiac hepcidin/ferroportin axis is essential for the cell-autonomous control of the intracellular iron pool upon which normal cardiac function depends [43].

The cardiac hepcidin/ferroportin axis also protects the heart in the setting of systemic iron deficiency. Indeed, animals with hepcidin-deficient hearts developed a greater hypertrophic response to sustained dietary iron restriction than their littermate controls [43]. Cardiac hepcidin protein was upregulated rather than downregulated by dietary iron restriction in vivo and by iron chelation in vitro. That hepcidin regulation in the heart is divergent from that of the liver, suggests that it functions to counteract the effects of reduced systemic iron availability, by promoting iron retention within the cardiomyocyte.

Comparison between cardiac and ubiquitous mouse models of disrupted hepcidin/ferroportin axis further reveals that cardiomyocyte iron levels are a balance between local homeostasis, achieved through the cardiac hepcidin/ferroportin axis and systemic iron homeostasis, controlled by the systemic hepcidin/ferroportin axis. For instance, despite a marked increase in cardiac ferroportin levels, systemic loss of hepcidin responsiveness did not lead to the same cardiovascular dysfunction seen in mice with a cardiac-specific loss of hepcidin responsiveness [43]. This suggests that increased systemic availability can mitigate against the effects of uncontrolled iron release in cardiomyocytes. The interaction between the cardiac and systemic hepcidin/ferropotin axes in hemochromatosis patients is less clear. In particular, it remains to be established if the cardiac axis has a modifying effect on the degree of cardiac iron loading and ensuing cardiomyopathy, and whether differences in cardiac iron control between patients (e.g., due to factors such as local inflammation and ischemia) explain the lack of concordance between the severity of liver and of heart iron loading in a significant proportion of hemochromatosis and thalassemia major patients [47], [48].

## 4 Questions & implications

The finding that the cardiac hepcidin/ferroportin axis is essential for cardiomyocyte iron homeostasis and heart function poses a number of pertinent questions. Rat studies have shown that hepcidin RNA and protein are upregulated in both the ischemic portion of the infarcted heart and in the serum of infarcted animals 24 h after myocardial infarction MI [49]. In humans, hepcidin has been shown to be elevated in the serum following MI as early as 4 h, in a manner that is independent of serum iron levels and of inflammatory markers [50]. Therefore, it would important to interrogate the role of cardiac hepcidin in ischemic heart disease and in ischemia-reperfusion injury. Additionally, it remains to be established how inflammation, a hallmark of cardiovascular disease and a known regulator of hepcidin, impinges on the cardiac hepcidin/ferroportin axis, and on cardiomyocyte iron levels. In the context of hemochromatosis, it remains to be established how the systemic and cardiac hepcidin/ferroportin axes interact, and the extent to which local iron regulation in the heart mitigates against or exacerbates iron overload cardiomyopathy. Finally, greater understanding needs to be gained of the effects on cardiac iron homeostasis of hepcidin mimetics and inhibitors newly developed for the treatment of iron overload and iron deficiency respectively.

## Grants

S.L-L. is funded by a British Heart Foundation Intermediate Fellowship FS/12/63/29895.

## Author Contributions

S.L-L. drafted the manuscript and produced the figure.
